# Design of a Microwave Heating and Permittivity Measurement System Based on Oblique Aperture Ridge Waveguide

**DOI:** 10.3390/s23084035

**Published:** 2023-04-17

**Authors:** Mingyi Gou, Qian Chen, Penghao Dong, Changjun Liu, Kama Huang

**Affiliations:** 1 College of Electronics and Information Engineering, Sichuan University, Chengdu 610064, China; 2 Laboratory of Microwave Energy Applications, Yibin Industrial Technology Research Institute of Sichuan University, Yibin 644000, China

**Keywords:** permittivity, oblique aperture ridge waveguide, artificial neural network, microwave heating

## Abstract

In this paper, an oblique aperture ridge waveguide operating at 2450 MHz is proposed, and, using the ridge waveguide, a permittivity measurement system is constructed which can measure the permittivity of materials during microwave heating. The system calculates the amplitudes of the scattering parameters by using the forward, reflected and transmitted powers of the power meters, and it reconstructs the permittivity of the material by combining the scattering parameters with an artificial neural network. The system is used to measure the complex permittivity of mixed solutions of methanol and ethanol with different ratios at room temperature, and the permittivity of methanol and ethanol with increasing temperature, from room temperature to 50 °C. The measured results are in good agreement with the reference data. The system allows simultaneous measurement of the permittivity with microwave heating and provides real-time, rapid changes in the permittivity during heating, avoiding thermal runaway and providing a reference for applications of microwave energy in the chemical industry.

## 1. Introduction

There is a wide range of microwave applications in environmental science, medicine, chemistry, biology and our daily life [1,2,3,4,5,6,7,8]. These applications involve three main characteristics, i.e., microwave transmission, reflection and absorption in the medium [9,10,11], which are closely related to the complex permittivity of a medium. Complex permittivity can reflect the absorption, reflection and transmission ability of the medium to the electromagnetic waves during the interactions between the electromagnetic waves and the medium.

The permittivity of a material varies with changes in temperature. Due to variable permittivity, the microwave absorption ability of some materials can be changed during the microwave heating process, which may lead to the “thermal runaway” phenomenon, and even safety accidents. The emerging discipline of microwave chemistry has also flourished with the development of microwave heating. Microwaves can directly interact with a chemical system, and use its thermal effect to promote a variety of chemical reactions. The essence of chemical reactions is the renewal of substances with permittivity changing in the chemical reactions, and the permittivity real-time measurement of the reactants has become an important means of studying the principle of interactions between microwaves and reactants. Therefore, measurement of the complex permittivity during the microwave heating process is a prerequisite for the safe use of microwave energy.

Different permittivity measurement methods and systems have been proposed according to different physical forms of media, such as solid, liquid, and solid powder, and to different temperature environments. At present, the traditional measurement methods have been divided into two main categories: network parameter methods and resonant methods [12]. These methods both have their own advantages, but also have some limitations. In network parameter methods, the sensor and material are equivalent in a single or multi-port network that combines electromagnetic parameter measurement methods, such as the transmission/reflection method, free-space method, short-ended method, and open-ended method [13,14,15,16,17], and then its permittivity can be measured through a vector network analyzer and other instruments. Network parameter methods offer relatively high accuracy over a wide frequency band and require less sample preparation than resonant methods [18], and therefore, are suitable for the measurement of permittivity of high-loss materials. In resonant methods, electromagnetic parameters are measured by changing the resonant frequency and quality factor when a sample is inserted into a measurement cell [19,20]. Resonant methods have high measurement accuracy, but the measurement frequency is only a single point, which is widely used in the measurement of permittivity of low-loss materials [21]. In general, for low-loss material measurements, the accuracy is high when using resonant methods, which are commonly used in point frequency measurement [22]. For high-loss material measurements, network parameter methods are widely used. These methods have the advantages of large measurement bandwidth, low requirements for sample preparation and simple system structure.

In fact, it is difficult for devices to measure the permittivity of materials directly. In network parameter methods, the scattering parameters of the medium can be obtained from microwave measurements, and the relationship between the permittivity and the scattering parameters is complicated. Deep learning allows computing models composed of multiple processing layers to learn data representation with multiple abstract levels, and the models can learn very complex functions [23]. Deep learning has developed rapidly in recent years, especially the artificial neural network. Neural networks can be trained to map a set of inputs to a set of outputs, making a wide range of applications possible, such as electromagnetic inversion, resistivity data reconstruction and image recognition [24,25,26]. Therefore, in this study, we use an artificial neural network which is the main architecture for deep learning to obtain the permittivity through the scattering parameters.

In view of the above, in this paper, we propose an integrated microwave heating and complex permittivity measurement system based on deep learning with the transmission/reflection method. The scattering parameters of the system are calculated by collecting relevant powers from the microwave power meters, and the permittivity of the medium is reconstructed by combining with an artificial neural network algorithm. The system can measure the permittivity of liquid, solid, and solid powder, and simultaneously enables the measurement of the permittivity during microwave heating.

## 2. Materials and Methods

### 2.1. System Design

**Figure 1:**
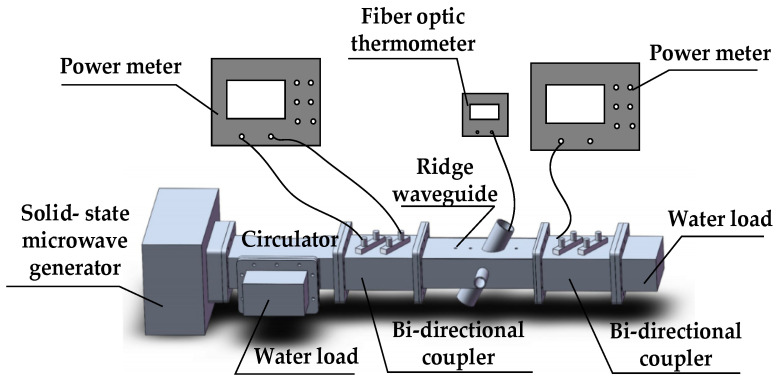
Permittivity measurement system at 2450 MHz.

The proposed permittivity measurement system using an oblique aperture ridge waveguide based on deep learning is shown in Figure 1. The system is mainly composed of a solid-state microwave generator, a circulator, two bi-directional couplers, an oblique aperture ridge waveguide, two water loads, two microwave power meters, and a fiber optic thermometer. For measurements, a quartz test tube containing the sample to be measured is placed in the ridge waveguide. Two bi-directional couplers are connected at both ends of the ridge waveguide. By connecting the microwave power meters to the bi-directional couplers, the forward and reflected power is measured at the input of the ridge waveguide, and the transmitted power can be obtained at the output port of the ridge waveguide. Microwave power generated by the solid-state microwave generator is used for microwave heating and the permittivity measurement of materials. In order to protect the microwave generator, a circulator with a water load is connected between the microwave generator and a bi-directional coupler. At the same time, a water load is also connected at the end of the system to absorb the microwave energy transmitted through the ridge waveguide. A fiber optic thermometer is placed into the material, and the temperature is monitored in real time. Finally, the measured forward power, reflected power and transmitted power are calculated as the corresponding scattering parameters, which are then used as the input of the artificial neural network to reconstruct the permittivity of the corresponding material.

### 2.2. Core Measurement Equipment Design

**Figure 2:**
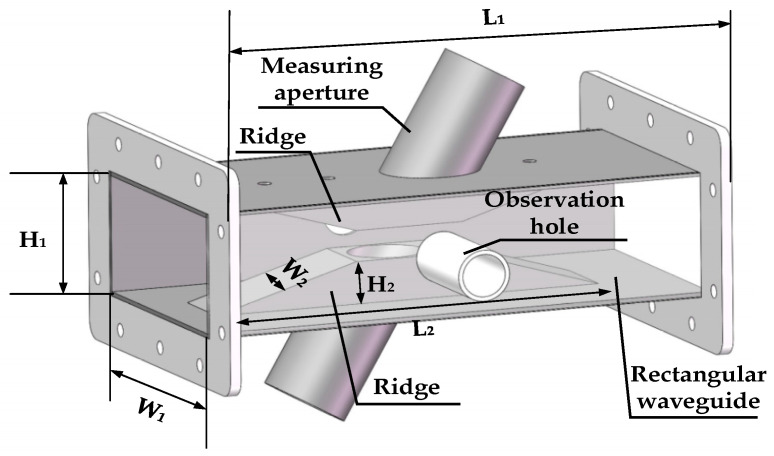
Schematic of the oblique aperture ridge waveguide.

The core equipment for measurements is designed at 2450 MHz, which is widely used in the chemical industry. The core device of the permittivity measurement system is an oblique aperture ridge waveguide, which consists of four parts: rectangular waveguide, ridge, measuring oblique aperture and observation hole, as shown in Figure 2. The waveguide length is L_1_, the waveguide width is W_1_, the waveguide height is H_1_, the ridge length is L_2_, the ridge width is W_2_ and the ridge height is H_2_. The size dimensions of the measuring device are listed in Table 1. The ridge waveguide is designed based on the standard rectangular waveguide BJ22, which bends the wide wall to form a ridge. Due to the ridge waveguide, the electromagnetic field is focused between the two ridges. The ridge waveguide has a hole in each of the two narrow walls, where the sample to be measured can be observed. To avoid leakage of electromagnetic energy, the observation holes extend outwards to form a circular waveguide, which operates in a cut-off state. The ridges of the broad walls have a measuring hole for the material to be measured. The circular waveguide formed from the measurement holes also operates in the cut-off state. The measurement holes form an oblique angle with the waveguide. This structure allows the sample to be located in the region with strong electric field, which improves the measurement sensitivity.

**Figure 3:**
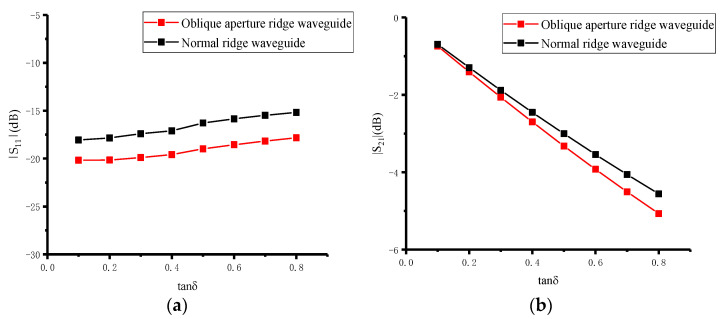
Sensitivity comparison results of oblique aperture ridge waveguide and normal ridge waveguide at  ε′ = 5: (**a**) Sensitivity comparison results of |*S*_11_|; (**b**) sensitivity comparison results of |*S*_21_|.

**Figure 4:**
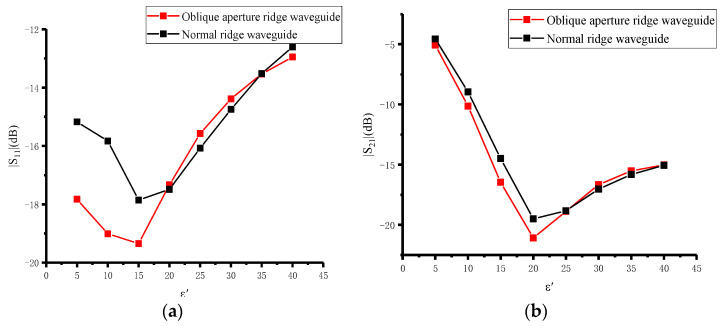
Sensitivity comparison results of oblique aperture ridge waveguide and normal ridge waveguide at tanδ = 0.8: (**a**) Sensitivity comparison results of |*S*_11_|; (**b**) sensitivity comparison results of |*S*_21_|.

When the real part of the permittivity of the material to be measured varies from 1 to 40 and the loss tangent varies from 0.1 to 0.8, the scattering parameters are simulated using full-wave simulations. The simulation results show that the scattering parameters, i.e., |*S*_11_| and |*S*_21_|, can reflect a change in the permittivity of the material when the real part is 1–40 and the loss tangent is 0.1–0.8. By using the scattering parameters, the permittivity of the material to be measured can be reconstructed. Figure 3 and Figure 4 show the simulation results for the oblique aperture and the normal ridge waveguide [27] with a real part of 5 and a loss tangent of 0.1–0.8, and a loss tangent of 0.8 and a real part of 1–40, respectively. Comparing the scattering parameters of the oblique aperture and normal ridge waveguide, the scattering parameters of the oblique aperture ridge waveguide have a greater range of variation and higher measurement sensitivity for the same range of variation in loss tangent and the real part of the permittivity.

### 2.3. Artificial Neural Network Design

**Figure 5:**
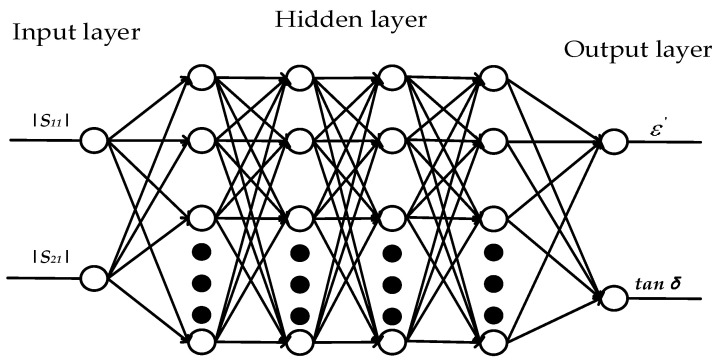
Neural network prediction model.

In this paper, the artificial neural network algorithm is used to reconstruct the complex permittivity. We used the artificial neural network to reconstruct the permittivity of the material by measuring the scattering parameters. The network is mainly composed of three parts: an input layer, four hidden layers and an output layer. The input vector of the input layer is the scattering parameters (|*S*_11_|, |*S*_21_|), and the output layer outputs two dielectric characteristic vectors (real part ε′, loss tangent tanδ), as shown in Figure 5. The output value of the layer $a^{i}$ (*i* = 1, 2, 3, 4, 5) is:

(1) $$a^{i}=f\left(\sum_{j-1}^{m}\omega^{i-1}a^{i-1}+b^{i-1}\right)$$

where m is the number of neurons of the (*i* − 1) layer, $\omega^{i-1}$ is the weight coefficient matrix of the (*i* − 1) layer, $a_{j}^{i-1}$ is the output value of the j neuron of the (*i* − 1) layer, $b^{i-1}$ is the bias vector of the (*i* − 1) layer, *f*(*x*) is the activation function. The input layer $a^{0}$ is the scattering parameter:

(2) $$\;a^{0}={\left[|S_{11}|,|S_{21}|\right]}^{T}$$

The output layer $a^{5}$ is the permittivity:

(3) $$a^{5}={\left[\varepsilon^{\prime },\tan\delta \right]}^{T}$$

**Figure 6:**
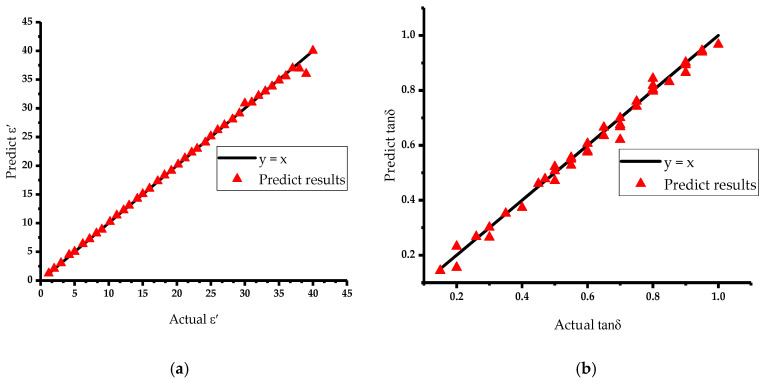
Permittivity prediction results: (**a**) Prediction effect of ε′; (**b**) prediction effect of tanδ.

A portion of the simulation data as training samples is selected to complete the training of the neural network. To verify the prediction effect of the neural network, 40 groups of sample data, not used for training, are selected and input into the trained neural network. The prediction results with high accuracy are shown in Figure 6, since the relationship between the predicted value and the true permittivity is close to the theoretical curve *y = x.* The predicted mean absolute errors of the real part of the permittivity and the loss tangent are 1.15% and 3.56%, respectively, indicating that the neural network model is highly reliable.

## 3. Results

### 3.1. Room Temperature Experiment

**Figure 7:**
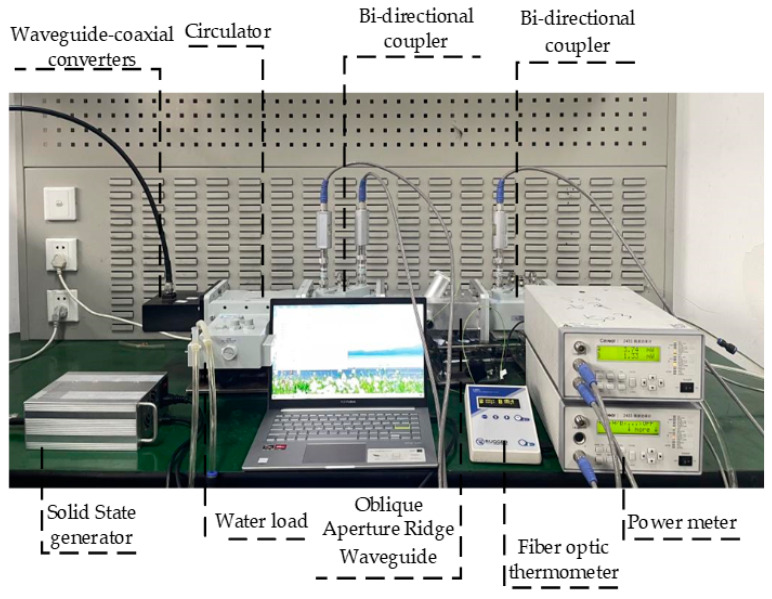
Photo view of the permittivity measurement system.

The reliability of the microwave heating and measurement system were verified at room temperature. Methanol and ethanol are widely used chemical reagents; therefore, we chose methanol and ethanol solutions and their mixtures for measurement at 2450 MHz frequency. During the experiments, the solution to be tested was poured into a quartz test tube and placed in the oblique aperture ridge waveguide. The forward, reflected and transmitted powers were measured using the microwave power meters, and then the amplitudes of *S*_11_ and *S*_21_ were calculated from these powers. The whole measurement system was built according to Figure 1, and a photograph of the permittivity measurement system is shown in Figure 7.

The reference values of the complex permittivity of methanol and ethanol are 24.97 and 8.94, respectively. When methanol and ethanol are mixed in different ratios, the real part of their complex permittivity varies from 8.94 to 24.97. As calculated by Equation (4), the permittivity of the mixed solution gradually increases with the methanol concentration. Therefore, we prepared the mixed solution for measurement according to the equal proportional variation, and the measured values agreed with the reference values. 

The reference values for pure solutions were obtained from [28], while the complex permittivity of mixed solutions is ε*_e__ff_* = ε′ − *j*ε″, where ε′ represents the real part of the complex permittivity and ε″ represents the imaginary part of the complex permittivity. It can be calculated from the Bruggeman formula [29] as:

(4) $$\varepsilon_{eff}=\frac{1}{4}[(3\alpha -1)\varepsilon_{i}+(2-3\alpha )\varepsilon_{e}]+\frac{1}{4}{\left\{{\left[(1-3\alpha )\varepsilon_{i}+(3\alpha -2)\varepsilon_{e}\right]}^{2}+8\varepsilon_{i}\varepsilon_{e}\right\}}^{\frac{1}{2}}$$

In the formula, ε*_eff_* is the permittivity of the mixed solution, ε*_i_* is the permittivity of the blended solution, ε*_e_* is the permittivity of the base solution, and α is the volume ratio of the blended solution to the mixed solution. The reference values of the permittivity of mixed solutions were calculated according to the Bruggeman formula, and the volume ratio *α* was varied by 20% for each group.

The total volume of solution to be measured was 100 mL and six solutions were prepared. In the first solution, only 100 mL of ethanol was used, each subsequent solution was mixed by reducing the ethanol amount by 20 mL and increasing the methanol amount by 20 mL until the sixth solution was only 100 mL of methanol solution. At room temperature, the permittivity was measured by adding 5 mL of each solution into a test tube, and the average of the three experimental tests were taken as the measured permittivity of material. The results of the experiments are shown in Table 2 and Table 3.

It can be seen from the measurement results in Table 2 and Table 3 that the microwave heating and measurement system is capable of accurately measuring the complex permittivity of methanol, ethanol solvents and their mixtures at room temperature, which is consistent with the theoretical values in the references. The relative error of the real part of the complex permittivity measurement is within 10%, and the relative error of the loss tangent measurement is also within 7%. In this experiment, the measurement error in the real part of the solution’s complex permittivity was the largest when methanol and ethanol were mixed at a ratio of 2:3. The error could be due to the change in the dielectric properties of the solution caused by the poor mutual solubility of the methanol and ethanol reagents. These errors may have also been caused by the differences in temperature and humidity in the experimental environment compared to in the literature. In addition to the complex structure of the measurement system and systematic errors in the individual microwave components, the inversion algorithm model may lead to some minor errors.

### 3.2. Increasing Temperature Experiment

**Figure 8:**
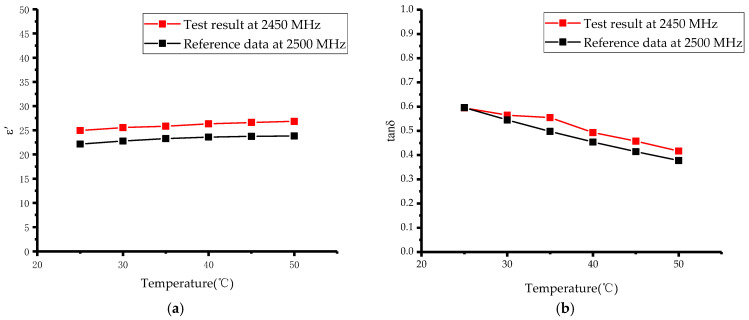
Variations in methanol complex permittivity with temperature: (**a**) ε′; (**b**) tanδ.

**Figure 9:**
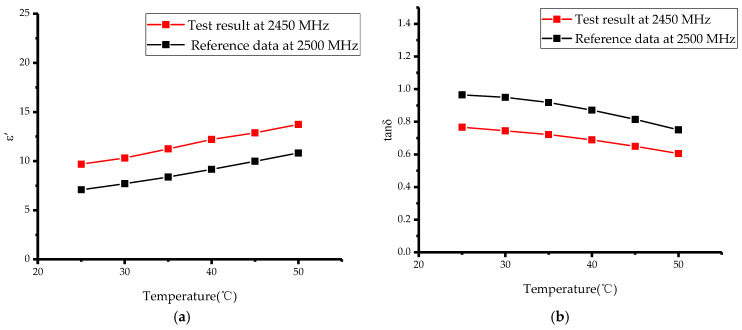
Variations in ethanol complex permittivity with temperature: (**a**) ε′; (**b**) tanδ.

After verifying the reliability of the system at room temperature, we further used the system to measure the permittivity of materials with increasing temperature during microwave heating. We chose methanol and ethanol as the materials to be tested in the warming experiment. The boiling point of methanol is 64.7 °C and that of ethanol is 78.3 °C. Considering the boiling points of the two organic reagents, the output power of the microwave generator was set to 10 W. The complex permittivity of the material was measured from room temperature to 50 °C, at 5 °C intervals. A fiber optic thermometer was used for real-time temperature monitoring. The forward, reflected and transmitted power at each temperature point were recorded to calculate the scattering parameters by using the power meters, and then the permittivity was reconstructed by using an artificial neural network. The measurement results are shown in Figure 8 and Figure 9.

Measurement of the complex permittivity of methanol and ethanol under increasing temperature conditions were compared with the results in [30]. Figure 8 and Figure 9 show that the results of this experiment at 2450 MHz have the same trend as the reference results at 2500 MHz, with only small differences in the specific values. This may be due to the fact that there is a deviation in frequency between the two experiments and the permittivity varies with the frequency, hence, the small deviation in the tested values at the two frequencies. In addition, there may be some minor errors in the purity of the organic reagents as well as in network of inversion algorithms, which can also lead to deviations in the measured values, but the accuracy of the measurements are not affected. In summary, we experimentally verified the accuracy and reliability of this microwave heating and complex permittivity measurement system. Moreover, we designed the system to use a quartz tube as a container for the material to be measured, thus, avoiding direct contact between the material and the measuring device, and allowing the measurement of the complex permittivity of corrosive materials.

## 4. Discussion

In this paper, a system for microwave heating and measurement of the complex permittivity was designed based on the transmission/reflection method, and it was experimentally verified that the system could achieve rapid and accurate measurement of the complex permittivity during microwave heating. The permittivity of methanol and ethanol and their mixed solutions were measured at room temperature. In addition, the permittivity of microwave-heated methanol and ethanol solutions were measured during the process of increasing temperature, and the results were in agreement with the references, indicating that the system could be used to measure the permittivity of a material at high temperatures. In general, the comparison of the experimental data with the simulation results showed good agreement. The small errors between the two results could be due to system errors introduced by coaxial cables and other devices, minor measurement errors in the power meters, inversion errors introduced by the artificial neural network algorithm and so on.

During the process of microwave heating and drying of materials, the rapid increase in temperature that some substances experience can lead to sudden changes in the permittivity of the substances. By measuring the permittivity of a substance under microwave heating conditions, the real-time variation of the substance’s permittivity with temperature can be obtained. In addition, a rapid change in permittivity can cause the absorption of microwave energy to increase rapidly, forming a positive feedback, resulting in “thermal runaway”. The system provides a method to measure the complex permittivity during microwave heating, which can obtain the change rule of the complex permittivity during the heating process in real time and control the microwave power in real time. By using the proposed microwave heating and permittivity measurement system, thermal runaway can effectively be avoided, providing safety guidance and support for applications of microwave energy.

## Figures and Tables

**Table 1:** Structure dimension table of the oblique aperture ridge waveguide.

Parameter	Values (mm)
L_1_	237
W_1_	109.22
H_1_	54.61
L_2_	180
W_2_	34
H_2_	20

**Table 2:** Real part of the permittivity of solutions.

Media (Volume Ratio)	Reference ε′	Measurement ε′	Errors (%)
Methanol:Ethanol = 0:5	8.94	9.69	+8.4%
Methanol:Ethanol = 1:4	11.26	12.06	+7.1%
Methanol:Ethanol = 2:3	14.09	15.48	+9.8%
Methanol:Ethanol = 3:2	17.37	17.36	−0.06%
Methanol:Ethanol = 4:1	21.03	20.79	−1.1%
Methanol:Ethanol = 5:0	24.97	24.96	−0.04%

**Table 3:** Loss tangent of solutions.

Media (Volume Ratio)	Reference tanδ	Measurement tanδ	Errors (%)
Methanol:Ethanol = 0:5	0.85	0.80	−5.9%
Methanol:Ethanol = 1:4	0.78	0.74	−5.1%
Methanol:Ethanol = 2:3	0.72	0.71	−1.4%
Methanol:Ethanol = 3:2	0.66	0.70	+6.1%
Methanol:Ethanol = 4:1	0.62	0.63	+1.6%
Methanol:Ethanol = 5:0	0.58	0.59	+1.7%

## Data Availability

The data that support the findings of this study are available from the corresponding author upon reasonable request.

## References

[1] Rodriguez-Morales F., Occhiogrosso V., Arnold E. (2021). Multichannel UWB microwave radar front-end for fine-resolution measurements of terrestrial snow cover. Proceedings of the 2021 International Conference on Radar, Antenna, Microwave, Electronics, and Telecommunications (ICRAMET).

[2] David N., Liu Y., Kumah K.K., Hoedjes J.C., Su B.Z., Gao H.O. (2021). On the power of microwave communication data to monitor rain for agricultural needs in Africa. Water.

[3] Han Z., Li Y., Luo D.-H., Zhao Q., Cheng J.-H., Wang J.-H. (2021). Structural variations of rice starch affected by constant power microwave treatment. Food Chem..

[4] Mansoori A., Isleifson D., Desmond D., Stern G. (2020). Development of Dielectric Measurement Techniques for Arctic Oil Spill Studies. Proceedings of the 2020 IEEE International Symposium on Antennas and Propagation and North American Radio Science Meeting.

[5] Kossenas K., Podilchak S.K., Comite D., Re P.D.H., Goussetis G., Pavuluri S.K., Griffiths S.J., Chadwick R.J., Guo C., Bruns N. (2021). A methodology for remote microwave sterilization applicable to the coronavirus and other pathogens using retrodirective antenna arrays. IEEE J. Electromagn. RF Microw. Med. Biol..

[6] Chen J., Zhu J., Xu W., Chen Y., Zhou J. (2022). Highly efficient H_2_ and S production from H_2_S decomposition via microwave catalysis over a family of TiO_2_ modified Mo_x_C microwave catalysts. Fuel Process. Technol..

[7] Mohamed B.A., Bilal M., Salama E.S., Periyasamy S., Fattah I.M.R., Ruan R., Awasthi M.K., Leng L. (2022). Phenolic-rich bio-oil production by microwave catalytic pyrolysis of switchgrass: Experimental study, life cycle assessment, and economic analysis. J. Clean. Prod..

[8] Guzik P., Kulawik P., Zając M., Migdał W. (2022). Microwave applications in the food industry: An overview of recent developments. Crit. Rev. Food Sci. Nutr..

[9] Harid V., Kim H., Li B.Z., Lei T. (2023). A method for non-destructive microwave focusing for deep brain and tissue stimulation. PLoS ONE.

[10] Ochi H., Shimamoto S., Liu J., Yamaoka Y. (2021). Non-contact Blood Pressure Estimation with Pulse Wave employing Microwave Reflection. Proceedings of the 2021 IEEE International Conference on Communications Workshops (ICC Workshops).

[11] Choe H.-S., Lee J.-S., Kweon J.-H., Nam Y.-W., Choi W.-H. (2022). High-performance microwave absorption heating honeycomb sandwich composite with electroless nickel-plated glass fiber. Compos. Struct..

[12] Hosseini M.H., Heidar H., Shams M.H. (2016). Wideband nondestructive measurement of complex permittivity and permeability using coupled coaxial probes. IEEE Trans. Instrum. Meas..

[13] Kalisiak M., Wiatr W., Papis R. (2022). Design of a Waveguide Test Cell for Q Band Liquid Permittivity Measurements. Proceedings of the 2022 24th International Microwave and Radar Conference (MIKON).

[14] Lu Z.H., Zheng M.X., Chen G.P., Xu J.H., Huang G.L. (2022). Planar Scanning Measurement System of Material Properties Based on Free Space Method. Proceedings of the 2022 IEEE Conference on Antenna Measurements and Applications (CAMA).

[15] Park Y.J. (2009). Short-ended coaxial cylinder probe measuring bulk dielectric constant using TDR. Microw. Opt. Technol. Lett..

[16] Neumayer M., Flatscher M., Bretterklieber T. (2019). Coaxial Probe for Dielectric Measurements of Aerated Pulverized Materials. IEEE Trans. Instrum. Meas..

[17] Dilman I., Akinci M.N., Yilmaz T., Çayören M., Akduman I. (2022). A Method to Measure Complex Dielectric Permittivity With Open-Ended Coaxial Probes. IEEE Trans. Instrum. Meas..

[18] Hasar U.C. (2008). A new calibration-independent method for complex permittivity extraction of solid dielectric materials. Electronics.

[19] Li C., Wu C., Shen L. (2022). Complex Permittivity Measurement of Low-Loss Anisotropic Dielectric Materials at Hundreds of Megahertz. Electronics.

[20] Chen Q., Long Z., Shinohara N., Liu C.J. (2022). A substrate integrated waveguide resonator sensor for dual-band complex permittivity measurement. Processes.

[21] Bartley P.G. (2022). Permittivity Measurement of Low-Loss Materials using Embedded Resonance. Proceedings of the 2022 IEEE International Instrumentation and Measurement Technology Conference (I2MTC).

[22] Kik A. (2016). Complex permittivity measurement using a ridged waveguide cavity and the perturbation method. IEEE Trans. Microw. Theory Tech..

[23] Lecun Y., Bengio Y., Hinton G. (2015). Deep learning. Nature.

[24] Puzyrev V. (2019). Deep learning electromagnetic inversion with convolutional neural networks. Geophys. J. Int..

[25] Liu B., Guo Q., Li S., Liu B., Ren Y., Pang Y., Guo X., Liu L., Jiang P. (2020). Deep learning inversion of electrical resistivity data. IEEE Trans. Geosci. Remote Sens..

[26] El Ogri O., Karmouni H., Sayyouri M., Qjidaa H. (2021). 3D image recognition using new set of fractional-order Legendre moments and deep neural networks. Signal Process. Image Commun..

[27] Tan Q., Zhu H.C., Ma W.Q., Yang Y., Huang K.M. (2019). High temperature dielectric properties measurement system at 915 MHz based on deep learning. Int. J. RF Microw. Comput. Aided Eng..

[28] Chen Q., Huang K.M., Yang X., Luo M., Zhu H. (2011). An artificial nerve network realization in the measurement of material permittivity. Prog. Electromagn. Res..

[29] Nelson S.O. (1992). Measurement and applications of dielectric properties of agricultural products. IEEE Trans. Instrum. Meas..

[30] Gregory A.P., Clarke R. (2012). Tables of the Complex Permittivity of Dielectric Reference Liquids at Frequencies up to 5 GHz.

